# Extreme hyperthermia tolerance in the world’s most abundant wild bird

**DOI:** 10.1038/s41598-020-69997-7

**Published:** 2020-08-04

**Authors:** M. T. Freeman, Z. J. Czenze, K. Schoeman, A. E. McKechnie

**Affiliations:** 1 South African Research Chair in Conservation Physiology, South African National Biodiversity Institute, Pretoria, South Africa; 2 DSI-NRF Centre of Excellence at the FitzPatrick Institute, Department of Zoology and Entomology, University of Pretoria, Pretoria, South Africa

**Keywords:** Ecophysiology, Animal physiology

## Abstract

The thermal tolerances of vertebrates are generally restricted to body temperatures below 45–47 °C, and avian and mammalian critical thermal maxima seldom exceed 46 °C. We investigated thermoregulation at high air temperatures in the red-billed quelea (*Quelea quelea*), an African passerine bird that occurs in flocks sometimes numbering millions of individuals. Our data reveal this species can increase its body temperature to extremely high levels: queleas exposed to air temperature > 45 °C increased body temperature to 48.0 ± 0.7 °C without any apparent ill-effect, with individual values as high as 49.1 °C. These values exceed known avian lethal limits, with tolerance of body temperature > 48 °C unprecedented among birds and mammals.

## Introduction

Survival and reproduction in hot environments are constrained by the upper limits to organisms’ thermal tolerances. Under high environmental heat loads, the avoidance of lethal body temperature (*T*_b_) drives fundamental behavioral trade-offs between thermoregulation and activities such as foraging^1,2^, and constraints on the evolution of upper thermal limits have important consequences for predicting responses to climate change^3^. Upper thermal limits also constrain performance under conditions of high metabolic heat production^4^ in contexts that include livestock production and food security under hotter future conditions^5,6^.

Body temperatures (*T*_b_) of vertebrates are thought to be limited to below 45–47 °C by the thermal sensitivity of cellular macromolecules^7–10^ and oxygen supply limitation^11,12^. Among terrestrial vertebrates, critical thermal maxima for squamate reptiles, rodents and birds are usually below 46 °C^13–16^. The same is generally true of maximum *T*_b_ values observed in birds, rodents and small bats in studies involving acute heat exposure but where critical thermal maxima were not quantified^17–20^. Typical lethal avian *T*_b_ values are 46.2–47.7 °C in two species of towhees^21^ and 46–47.8 °C in barred-rock chickens^16^, although the latter author reported lethal values as high as 48.8 °C associated with tracheal administration of 100% oxygen.

However, *T*_b_ above the typical vertebrate range has occasionally been documented. Critical thermal maxima above 47 °C have been reported in a small number of desert lizards (reviewed by Clusella-Trullas et al.^13^), with a value of 51.0 °C observed in ten adult *Aspidoscelis sexlineata*^22^. Among birds, three variable seed-eaters (*Sporophila aurita*), a passerine from the humid lowlands of Panama, survived *T*_b_ = 46.8–47.0 °C without any apparent ill-effects^23^. In a pioneering study of the use of surgically-implanted transmitters to measure avian *T*_b_, Southwick^24^ recorded *T*_b_ = 47.7 °C in a single white-crowned sparrow (*Zonotrichia leucophrys gambelli*). However, cloacal *T*_b_ measured simultaneously was 44.1 °C, and the 3.6 °C difference between this pair of measurements was the largest reported in the study^24^.

As part of a study of adaptive variation in avian heat tolerance, we investigated thermoregulation during acute heat exposure in the red-billed quelea (*Quelea quelea*). This small (18-g) African passerine is widely considered the most abundant non-domesticated bird on Earth, with post-breeding population estimates of ~ 1.5 billion individuals^25^. It is highly gregarious and forms huge flocks that may consist of several million individuals^26^. The peculiar natural history of this species led us to hypothesize that its thermal physiology differs from that of typical small songbirds. Red-billed queleas drink regularly^26^. However, the timing of flocks’ visits to water sources is presumably determined by the average hydration status of large numbers of flock members rather than that of single individuals. Under conditions where hydration status potentially varies substantially across individuals within a vast flock, selection should favour the capacity for water conservation via facultative hyperthermia. Accordingly, we predicted pronounced facultative hyperthermia buffers individual queleas from dehydration risk. To test this prediction, we quantified relationships between body temperature, evaporative heat loss and metabolic heat production in red-billed queleas in South Africa.

## Methods

All experimental procedures were approved by the University of Pretoria’s Animal Ethics Committee (NAS181/2019) and the Research Ethics and Scientific Committee of the South African National Biodiversity Institute (SANBI NZG/RES/P19/13) and birds were captured under permit JM 8,057/2019 from the Free State province’s Department of Economic, Small Business Development, Tourism and Environmental Affairs. The methods we used for quantifying the upper limits of evaporative cooling capacity and heat tolerance followed those of a recent series of studies of avian heat tolerance^27–30^.

### Study site and species

We trapped 20 red-billed queleas (body mass = 17.94 ± SD 1.19 g) using mist nets in agricultural fields near the town of Harrismith in South Africa (28° 06′ S, 29°10′E, 1754 m asl) during November 2019 (early austral summer). After capture, birds were transported by road (approximately 20-min trip) in cloth bags to a field laboratory, where they were held in cages (600 × 400 × 400 mm) for 1–16 h with ad libitum access to water and wild bird seed. Food was removed at least one hour prior to gas exchange and body temperature measurements, allowing individuals to habituate and ensure they were post-absorptive^31^.

### Air and body temperature measurements

Body temperature was measured using a temperature-sensitive passive integrated transponder (PIT) tag (Biotherm 13, Biomark, Boise, ID, USA) injected intraperitoneally in each bird. Prior to injection, all PIT tags were calibrated in a circulating water bath (model F34, Julabo, Seelbach BW, DE) over temperatures ranging 35 to 50 °C against a thermocouple meter (TC-1000, Sable Systems, Las Vegas, NV, USA), the output of which was verified against a mercury-in-glass thermometer with NIST-traceable accuracy before and after the PIT tag calibration. Temperatures measured by PIT tags deviated by 0.28 ± 0.23 °C (n = 23) from actual values and we corrected all measured values accordingly. Data from the PIT tags were recorded using a reader and transceiver system (HPR + , Biomark, Boise ID, USA). To measure air temperature during the gas exchange measurements, we inserted a thermistor probe (TC-100, Sable Systems, Las Vegas, NV, USA) through a hole sealed with a rubber grommet in the side of each metabolic chamber.

### Gas exchange measurements

An open flow-through respirometry system was used to measure evaporative water loss (EWL) and carbon dioxide production (${\dot{V}}_{{CO}_{2}}$) during measurements. Queleas were placed individually in 3-L (approximate dimensions 20 cm high × 15 cm wide × 10 cm deep) plastic chambers, previously shown to not absorb water vapour^27^, equipped with a mesh platform ~ 10 cm above a 1-cm layer of mineral oil into which excreta fell to prevent evaporation. The chambers were placed in a ~ 100 L ice chest modified such that temperature inside the chest was regulated using a Peltier device (AC-162 Thermoelectric Air Cooler, TE Technology, Traverse City MI, USA) controlled via a digital controller (TC-36–25-RS485 Temperature Controller, TE Technology, Traverse City MI, USA).

Atmospheric air supplied by an oil-free compressor was scrubbed of water vapour using a membrane dryer (Champion CMD3 air dryer and filter, Champion Pneumatic, Quincy IL, USA). The dried air was split into an experimental and baseline channel. A mass flow controller (Alicat Scientific Inc., Tuscon AZ, USA), calibrated using a soap-bubble flow meter (Gilibrator 2, Sensidyne, St Petersburg, FL, USA), regulated experimental flow rates to the animal chamber. The flow rate of the baseline channel was controlled using a needle valve (Swagelok, Solon, OH, USA). Within each chamber, the air inlet was positioned close to the lid with an elbow joint facing upwards (to minimize any potential convective cooling at higher flow rates) and the air outlet below the mesh platform to maximize air mixing. We used flow rates of 10.1–18.3 L min^−1^, depending on air temperature and individual behaviour, with flow rate regularly adjusted during measurements to maintain chamber humidity below a dewpoint of − 7.7 °C.

A respirometry multiplexer (model MUX3-1,101-18 M, Sable Systems, Las Vegas, NV) in manual mode and an SS-3 Subsampler (Sable Systems) sequentially subsampled excurrent air from the chamber and baseline air. Subsampled air was pulled through a CO_2_/H_2_O analyzer (model LI-840A, LI-COR, Lincoln, NE, USA), which was regularly zeroed using nitrogen and spanned for CO_2_ using a certified calibration gas with a known CO_2_ concentration of 1900 ppm (AFROX, Johannesburg, South Africa). The H_2_O sensor of the Li-840A was regularly zeroed using nitrogen and spanned using a dewpoint generator (DG-4, Sable Systems, Las Vegas NV). Voltage outputs from the analyzers and thermistor probes were digitized using an analog–digital converter (model UI-3, Sable Systems) and recorded with a sampling interval of 5 s using Expedata software (Sable Systems). All tubing in the system was Bev-A-Line IV tubing (Thermoplastic Processes Inc., Warren, NJ, USA).

### Experimental protocol

Measurements occurred during the day, and we quantified relationships between body temperature, metabolic heat production and evaporative heat dissipation over air temperatures of 28–52 °C by exposing birds to the same stepped air temperature profile involving 4-°C increments below 40 °C and 2-°C increments above 40 °C as used in previous studies^27–30^. Measurements commenced with a baseline air subsample until water and CO_2_ readings were stable (5 min). Birds spent a minimum of 10 min at each air temperature, with stable average values over the last 5 min at each air temperature value included in subsequent analyses, followed by another 5 min baseline. This approach to quantifying physiological responses to heat exposure is functionally analogous to the sliding cold exposure protocol used to elicit maximum metabolic rates during cold exposure^32^.

During measurements, individuals were continuously monitored using a video camera with an infrared light source. Only data from birds that remained calm during measurements (i.e., no sign of agitation or sustained escape behavior) were included in analyses. Trials were terminated and individuals immediately removed from the chamber when a bird reached its thermal endpoint characterized by sustained escape behaviour (i.e., agitated jumping) or a loss of coordination or balance, often associated with a sudden decrease in EWL or resting metabolic rate. Individual critical thermal maximum was taken as the body temperature associated with the onset of loss of balance and or uncoordinated movement. Immediately after each bird was removed from the chamber, its belly feathers were dabbed with 80% ethanol to accelerate heat loss and it was placed in a recovery cage with ad libitum water and food. Each bird was later released at the site of capture. This experimental protocol has been used previously for multiple species and, in one study with opportunistic monitoring for several weeks post-release, no adverse effects were observed^33^.

### Data analysis

We corrected for analyzer drift and lag using the relevant algorithms in Expedata software (Sable Systems, Las Vegas NV, USA). Eqs. 9.5 and 9.6 from Lighton^34^ were used to calculate ${\dot{V}}_{{CO}_{2}}$ and EWL from the lowest stable 5-min periods of CO_2_ and water vapour at a given air temperature*,* assuming 0.803 mg H_2_O mL^− 1^ vapour. As individuals were likely post-absorptive, we estimated resting metabolic rate from ${\dot{V}}_{{CO}_{2}}$ assuming respiratory exchange ratio (RER) = 0.71 and converted rates of ${\dot{V}}_{{CO}_{2}}$ to metabolic rate (W) using 27.8 J ml^−1^ CO_2_^35^. Rates of EWL were converted to rates of evaporative heat loss (EHL, W) assuming a latent heat of vaporization of water of 2.406 J mg^−1^ at 40 °C^36^. Body temperatures, rates of EWL and resting metabolic rates at thermoneutral air temperatures (Supplementary Fig. S1) were considered normothermic values.

All analyses were conducted in R 3.5.2^37^. Relationships between physiological response variables and air temperature as a predictor were analyzed using linear mixed-effects models (“lme” command) in the R package *nlme* 3.1–140^38^ after using *segmented* 1.1–0 ^39^ to identify inflection points. We accounted for pseudoreplication (multiple measurements per individual) by including individual identity as a predictor (random factor) in all analyses. We assessed significance at *p* < 0.05 and values are presented as mean ± s.d.

## Results

**Figure 1:**
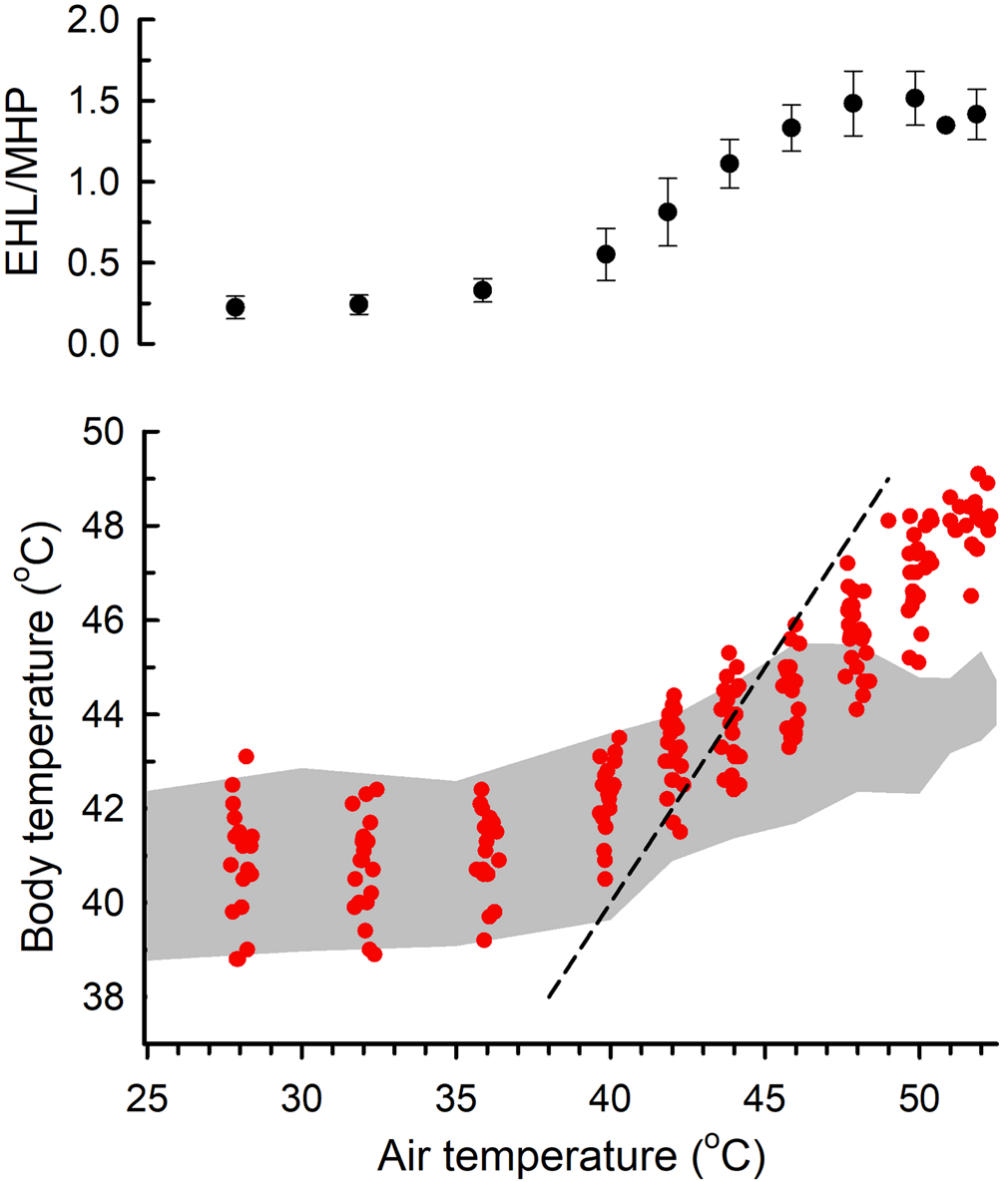
During acute heat exposure, the body temperature of red-billed queleas (*Quelea quelea*, red circles, lower panel) remained largely within the range reported in other passerine birds at air temperatures below 45 °C but increased well above previously-documented values at higher air temperatures. The grey band is the range of individual values in five Australian species^40^ and three southern African species^27^ in studies using the same experimental protocol. The dashed line indicates equality between air and body temperatures. The ratio of evaporative heat loss (EHL) to metabolic heat production (MHP) increased to an average maximum value of 1.49 at air temperatures above 46.9 °C (upper panel).

The normothermic body temperature of queleas was 40.9 ± 0.9 °C (n = 20), a value typical for small passerines (Fig. 1). Above an inflection air temperature of 38.0 ± SE 0.6 °C, body temperature increased by 0.5 °C per 1 °C increase in air temperature. Body temperature reached an estimated critical thermal maximum (i.e., maximum values associated with a loss of coordination and motor function) of 48.0 ± 0.7 °C (n = 20) at an air temperature of 50.9 ± 1.5 °C. Individual maximum values were 46.4—49.1 °C, with 75% of individuals reaching body temperature ≥ 48.0 °C (Fig. 1). Concurrent measurements of metabolic heat production (MHP) and evaporative heat loss (EHL) (Supplementary Fig. S1) revealed that EHL/MHP reached a maximum value of 1.49 at air temperature > 46.9 ± SE 0.5 °C (Fig. 1), confirming the queleas’ maximum evaporative cooling capacity had been attained.

## Discussion

**Figure 2:**
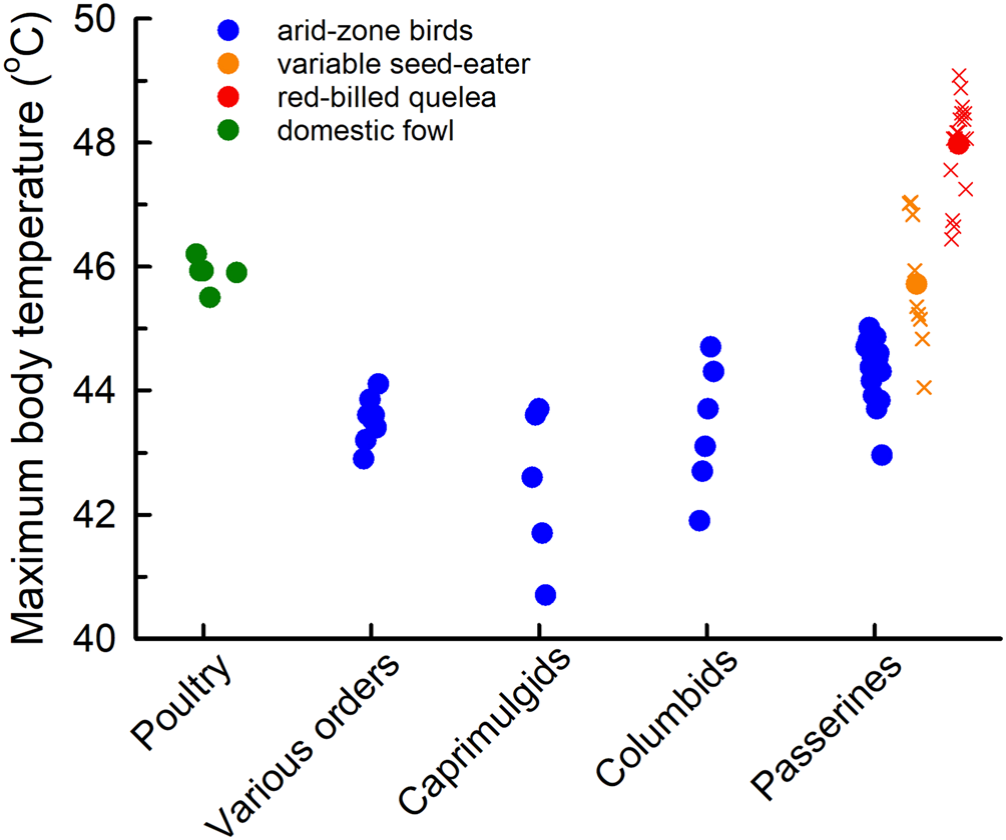
Maximum body temperatures attained during acute heat exposure in red-billed queleas (*Quelea quelea*) exceeded by a substantial margin those previously reported for birds. Species averages (in the case of domestic fowls, averages for breeds) are indicated using filled circles. Data for poultry are from^15,16^, and data for non-domesticated species from^27–30,40,41,50–54^. For variable seed-eaters (*Sporophila aurita*, data from^23^) and red-billed queleas (present study), both species averages (filled circles) and individual values (crosses) are shown.

Patterns of *T*_b_ during acute heat exposure supported our prediction that red-billed queleas have a pronounced capacity to tolerate hyperthermia. The species’ critical thermal maximum is substantially higher than the known avian range (Fig. 2), exceeding by 2–3 °C the values associated with breakdown of respiratory function in poultry^15,16^ and the body temperatures associated with loss of motor function in wild birds^27–30,40,41^. Moreover, the body temperature range tolerated by the queleas exceeds known avian lethal values for passerines^21^ and domestic fowls^15,16^. Tolerance of body temperature > 48 °C is unprecedented among birds and mammals, with higher values having been reported only in ectothermic vertebrates^13^ and invertebrates^42^.

The methods we used here to establish the upper limits of queleas’ heat tolerance and evaporative cooling capacity are identical to those of recent studies involving ~ 55 bird species, including three arid-zone representatives of the Ploceidae^27^, the family to which *Q. quelea* belongs. Extreme hyperthermia tolerance comparable to that of the queleas appears to be absent among small passerines inhabiting arid regions where air temperature maxima may approach or exceed 50 °C^27,29,40^. That desert birds apparently lack the ability to tolerate comparably high body temperatures, despite strong selection for water conservation^43^, suggests there are substantial costs to such extreme hyperthermia tolerance. These costs could potentially be related the synthesis of heat shock proteins (HSPs) and interactions with stress responses via the modification of glucocorticoid receptor function^9,44^.

The capacity of queleas to dissipate evaporatively a maximum of ~ 150% of metabolic heat production is relatively modest for a passerine; among 30 species, maximum EHL/MHP was 1.75 ± 0.31 ^27,29,30,40,45^. Among arid-zone passerines, regular-drinking species are capable of greater fractional increases in EWL and have higher heat tolerance limits compared to non-drinking species^45^. Our finding here of modest evaporative cooling capacity accompanied by extreme hyperthermia tolerance in a regularly-drinking species raises the possibility that coevolution of thermal physiology and water-dependence follows a different trajectory in species that form large flocks. Our hypothesis that avian social systems involving large flocks are associated with selection for pronounced hyperthermia tolerance could be tested further in gregarious species inhabiting hot, arid climates, particularly Australian species such as budgerigars (*Melopsittacus undulatus*) or cockatiels (*Nymphicus hollandicus*).

Our findings reveal it is possible for birds to evolve short-term tolerance of very high body temperature. Moreover, they identify red-billed queleas as a model for future studies of the physiological and molecular bases of extreme hyperthermia tolerance. We speculate that this species’ ability to tolerate *T*_b_ as high as 48–49 °C arises from an array of anatomical and molecular mechanisms, including a well-developed *rete opthalmicum* to maintain brain temperature well below core *T*_b_^46–48^ and pronounced heat shock protein expression^44,49^. Understanding the processes underlying the queleas’ ability to tolerate *T*_b_ values lethal to other endotherms may, we suspect, prove useful for biotechnology aimed at developing greater heat tolerance in birds and other organisms.

## Supplementary information

Supplementary data.

Supplementary Figure 1.

## Data Availability

The data generated during this study are included in the Supplementary Information files accompanying this published article.
