# Supplementary material for: Extreme hyperthermia tolerance in the world’s most abundant wild bird
Source: Sci Rep. 2020 Aug 4;10:13098. doi: 10.1038/s41598-020-69997-7 (PMC7403380; doi:10.1038/s41598-020-69997-7)
Supplement: Supplementary file 2 — Supplementary Figure 1. [file 41598_2020_69997_MOESM2_ESM.docx]

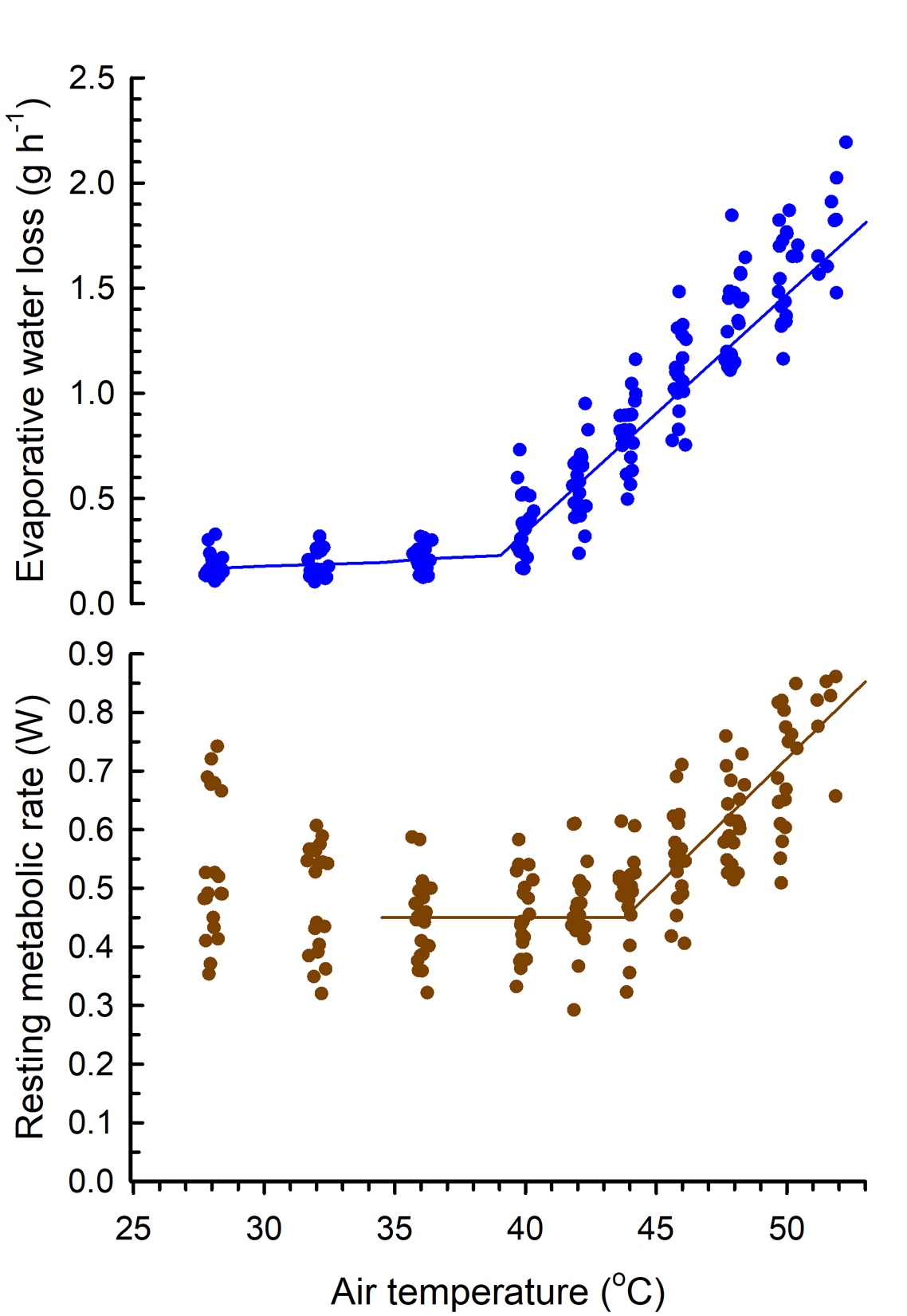


Figure S1. Relationships between air temperature and evaporative water loss (upper panel) and resting metabolic rate (lower panel) during acute heat exposure in red-billed queleas (*Quelea quelea*; n = 20). The solid lines are the relationships above and below inflection points from linear mixed-effects models that included individual as a random effect to account for multiple measurements per individual.
